# Supplementary material for: Genetic analysis of Thai cattle reveals a Southeast Asian indicine ancestry
Source: PeerJ. 2015 Oct 27;3:e1318. doi: 10.7717/peerj.1318 (PMC4627918; doi:10.7717/peerj.1318)
Supplement: Table S1 [file peerj-03-1318-s001.docx]

| Project code | Project name | Principal investigator | Reference URL |
| --- | --- | --- | --- |
| RDG5020077 | Native cattle production system and market pathway by farmers in the upper area of Bhumibol Dam | Dr. Kunya Tuntivisoottikul, King Mongkut Institute of Technology Ladkrabang | <http://elibrary.trf.or.th/>  project_content.asp?  PJID=RDG5020077 |
| RDG4920009 | Native cattle production of western region of Thailand | Dr. Sujate Cheunchom, Faculty of Agriculture, Kasetsart University, Kampangsan campus | <http://elibrary.trf.or.th/>  project_content.asp?  PJID=RDG4920009 |
| RDG4920007 | Native cattle production and market opportunity in northern region (Chaingmai, Prae, Lumpoon and Lumpang | Dr. Sompong Sruamsiri, Faculty of Animal Science, Maejo University | <http://elibrary.trf.or.th/>  project_content.asp?  PJID=RDG4920008 |
| RDG4920008 | Native cattle production: a case study in Ubonrachathani and Yasothorn | Mr. Somporn Duanyai, Faculty of Agriculture, Rajabhat University, Ubonrachathani | <http://elibrary.trf.or.th/>  project_content.asp?  PJID=RDG4920007 |
| RDG4920020 | Study on products and production system of Thai Native cattle of the the small holders in the upper North-East of Thailand (KhonKaen, Udonthanee and Kalasin) | Dr. Suthipong Uriyapongson, Department of Animal Science, Faculty of Agriculture, KhonKaen University | <http://elibrary.trf.or.th/>  project_content.asp?  PJID=RDG4920020 |
| RDG4520022 | Performance and carcass characteristics of Southern Thai Native cattle raised  under grazing system in Songkhla province | Dr. Chaiyawan Wattanachan, Faculty of Natural Resources, Prince of Songkhla University | <http://elibrary.trf.or.th/>  project_content.asp?  PJID=RDG4920021 |
| RDG5020078 | Native cattle production and market opportunity in Tak province | Dr. Thamrong Mekhora | <http://elibrary.trf.or.th/>  project_content.asp?  PJID=RDG5020078 |

S1 Table: List of researchers and projects who contributed bovine tissue samples to the “โครงการประสานงานวิจัยเรื่องการผลิตโคพื้นเมือง: ระบบการผลิตและโอกาสทางการตลาด”

(Coordination of research related to Thai native cattle production: Production system and market opportunity) under the project number RDC4920001 funded by the Thailand Research Fund (TRF). This project was coordinated by Dr. Yanin Opaspattanakit.
