# Supplementary figures and images for: Genetic analysis of Thai cattle reveals a Southeast Asian indicine ancestry
Source: PeerJ. 2015 Oct 27;3:e1318. doi: 10.7717/peerj.1318 (PMC4627918; doi:10.7717/peerj.1318)

**A**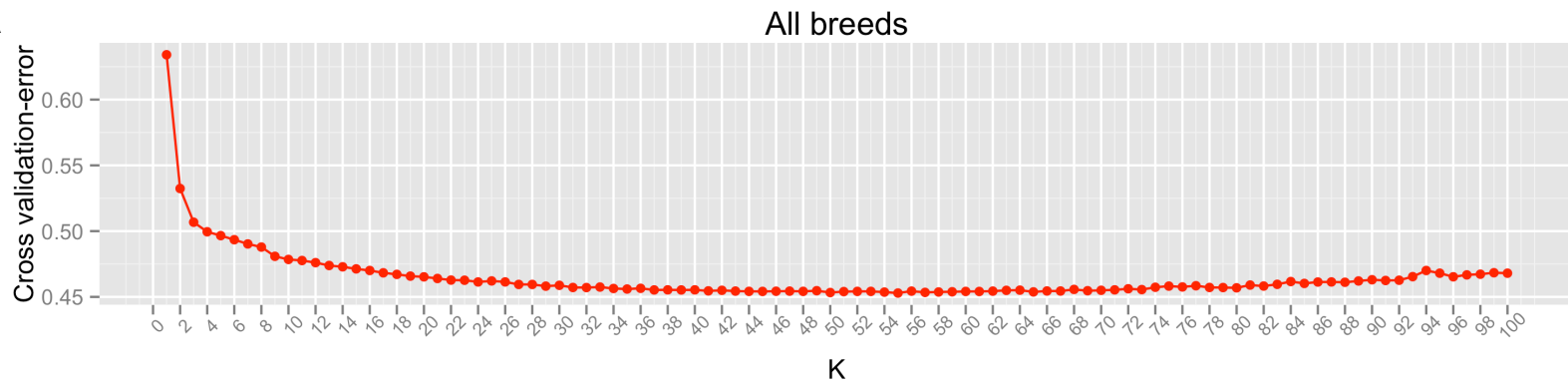**B**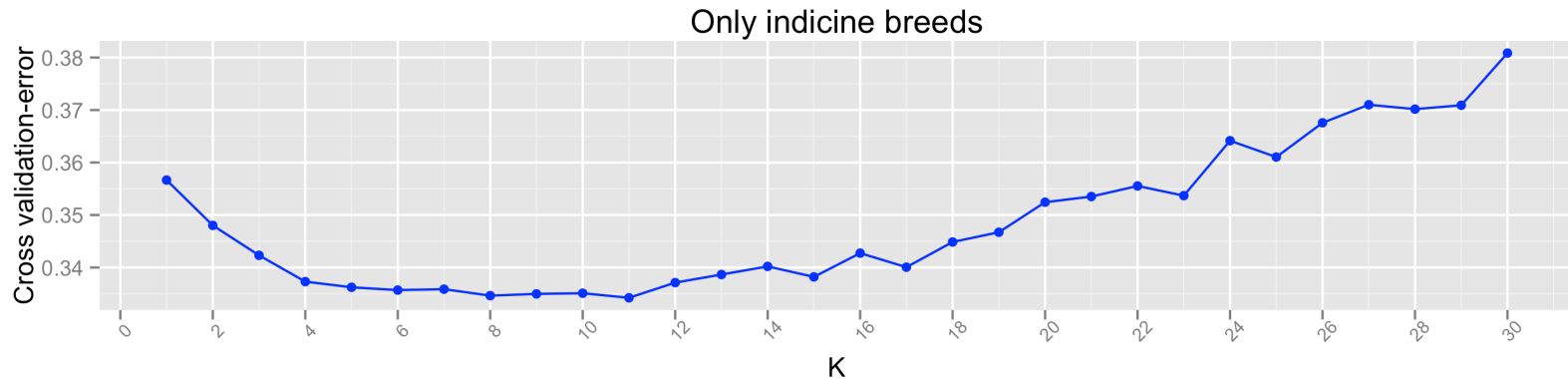**C**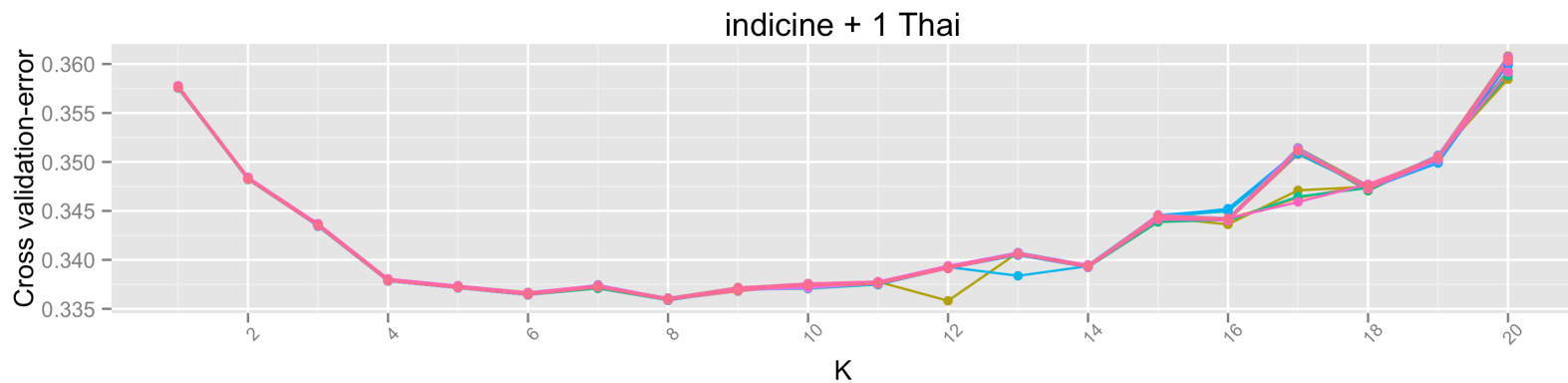

Supplement: Figure S1 — The cross-validation errors reported from ADMIXTURE for 10 cross-validation runs are shown from K = 2 up to K = 100. (A) The plot of ADMIXTURE cross-validation error at K = 2 to K = 100 of 1,397 cattle. (B) The plot of ADMIXTURE cross-validation error at K = 2 to K = 30 of 296 indicine cattle. (C) The combined plots of ADMIXTURE cross-validation errors from 28 ADMIXTURE experiments at K = 2 to K = 20 from 268 indicine cattle plus one Thai individual. [file peerj-03-1318-s006.pdf]

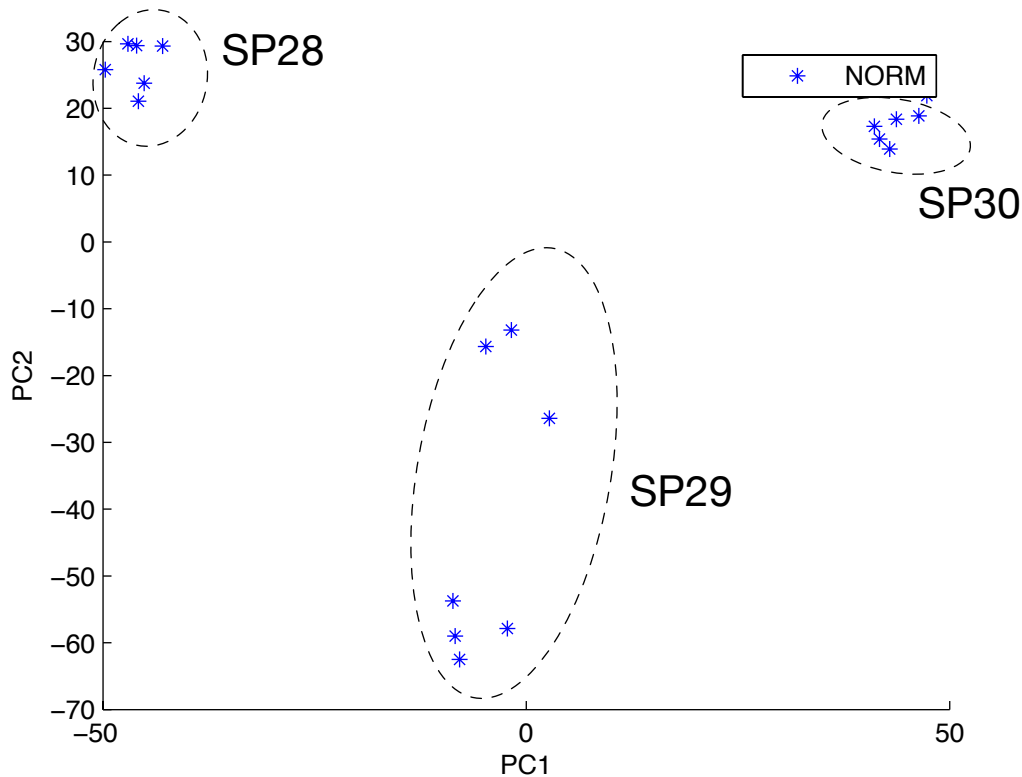

Supplement: Figure S2 [file peerj-03-1318-s008.pdf]
